# Triggers in functional motor disorder: a clinical feature distinct from precipitating factors

**DOI:** 10.1007/s00415-022-11102-1

**Published:** 2022-04-20

**Authors:** Christian Geroin, Jon Stone, Serena Camozzi, Benedetta Demartini, Marialuisa Gandolfi, Michele Tinazzi

**Affiliations:** 1 Neurology Unit, Movement Disorders Division, Department of Neurosciences, Biomedicine and Movement Sciences, University of Verona, Verona, Italy. P.le Scuro 10, 37134 Verona, Italy; 2 Centre for Clinical Brain Sciences, University of Edinburgh, Edinburgh, UK; 3 Physiotherapy Bachelor, University of Milan, Milan, Italy; 4 Department of Neurosciences, Biomedicine and Movement Sciences, University of Verona, Verona, Italy

**Keywords:** Functional neurological disorders, Functional motor disorders, Triggers, Precipitating factors, Diagnosis

## Abstract

**Background and objective:**

People with functional motor disorder (FMD) report triggers—sensory or motor-induced stimuli that exacerbate or initiate paroxysmal occurrences of their movement disorder. These are a distinct phenomenon from precipitating factors occurring at the initial onset of the disorder. We aimed to assess triggers in FMD and understand their relevance to paroxysmal variability often seen in FMD.

**Methods:**

We enrolled consecutive outpatients with a definite diagnosis of FMD. Each patient underwent a detailed clinical evaluation also including the presence of trigger factors and video-recordings both during neurological examination and physiotherapy treatment. Patients were classified as having “triggers” (T-FMD) or “not having triggers” (NoT-FMD) as well as “paroxysmal” compared to “persistent with paroxysmal variability”.

**Results:**

The study sample was 100 patients (82% female) with FMD; the mean age at onset was 41 years. Triggers were observed in 88% of patients and in 65 of these the FMD was pure paroxysmal. The most common triggers were movement or physical exercise, followed by emotional, visual, touch, and auditory stimuli; 39 (44%) were isolated and 49 (56%) were combined triggers. Among the T-FMD patients, FMD were paroxysmal in 74% (*n* = 65) and persistent with paroxysmal variability in 26% (*n* = 23). The T-FMD patients were younger (*p* = 0.016) and had a gait disorder (*p* = 0.035) more frequently than the NoT-FMD patients.

**Discussion:**

Triggers are frequent in FMD and may have diverse overlapping clinical presentations. In this sample, FMD was most often paroxysmal, suggesting the value of noting triggers as clinical clues in the diagnosis and rehabilitation of FMD.

**Supplementary Information:**

The online version contains supplementary material available at 10.1007/s00415-022-11102-1.

## Introduction

Patients with functional motor disorder (FMD), like those with other functional disorders, may report physical events (i.e., injury, illness, general anesthesia) or psychological trauma/stressors (i.e., panic) at the time of symptom onset**.** These are typically called precipitating factors [1, 2]. For instance, fixed dystonia [3] or functional paralysis is commonly reported after minor injury of the affected limb [4], functional seizures after panic attacks [5–8] and irritable bowel syndrome in patients with traveler’s diarrhea [9]. These precipitating events, reported either alone or together with a history of predisposing factors (i.e., other functional disorders or adverse experiences) [10] may contribute to abnormal attentional focus and alterations in “predictive processing” models of voluntary movement, ultimately leading to FMD [1, 11].

Separately it has been noted that some patients may be hypersensitive to external stimuli (i.e., visual, tactile, auditory) which may induce the sudden onset of or exacerbate FMD in individuals who *already have* the disorder. These triggers have been reported in patients with functional myoclonus [12] and with paroxysmal functional movement disorders [13].

Previous studies have also reported the presence of abnormal eye and cranial movements provoked by neurological examination (movement) [14, 15] and functional seizures induced by photic stimulation, saline injections and hypnosis [5]. Such triggers have been also described in other neurological diseases like migraine [16], epilepsy [17], and Parkinson’s disease [18], but they have never been extensively investigated and characterized in FMD. Identifying such triggers may help the clinician to make diagnosis of FMD in particular when they are paroxysmal and understanding them may be useful for therapeutic management of these disorders.

Therefore, in the present study we assessed the clinical features of triggers in a large sample of FMD patients and compared the clinical and demographic characteristics of patients with and without triggers to understand more about their role in FMD.

## Methods

In this observational cross-sectional study, we enrolled consecutive outpatients with a definite diagnosis of FMD attending for clinical evaluation and physiotherapy treatment at the Neurology Unit, Department of Neurosciences, Biomedicine and Movement Sciences, University of Verona (Italy).

### Patients

Patients were examined by a neurologist specialized in movement disorders and a “clinically definite” diagnosis of FMD was based on Gupta and Lang’s diagnostic criteria [19], response to distractibility maneuvers, demonstration of positive signs [20], and one or more clinical symptoms, including tremor, weakness, jerks, dystonia, gait disorders, parkinsonism and facial motor disorders [21]. Exclusion criteria were cognitive or physical impairments that impeded the patient from properly signing the informed consent form for participation in the study [21].

### Assessment

Each patient underwent a detailed clinical evaluation [21] and video-recording during both the neurological examination and physiotherapy treatment. We collected demographic, historical, and clinical details on age, sex, time between symptom onset and FMD diagnosis, FMD phenotype (weakness, gait disorders, tremor, dystonia, jerks, facial movement disorders, parkinsonism) [21], self-reported non-motor symptoms (fatigue, pain, headache, anxiety, insomnia depersonalization/derealization, panic attacks), specific neurological comorbidities documented by a neurologist’s diagnosis (polyneuropathy, hyperkinetic motor disorders, epilepsy, cerebrovascular diseases, migraine, multiple sclerosis, Parkinson’s disease and/or parkinsonism), and other (a free text was allowed). We also collected information on non-neurological comorbidities (hypertension, thyroid disease, arthritis and rheumatic disease, cancer, heart disease, dyslipidemia, diabetes mellitus and others), other functional neurological disorders (sensory functional symptoms, functional seizures [non-epileptic seizures], visual functional symptoms, cognitive functional symptoms, fibromyalgia, functional bowel syndrome). The questionnaire also recorded the presence of precipitating factors- physical or psychological events and surrounding circumstances which occurred at the onset of functional movement disorder [2, 22]—such as surgery, physical trauma, psychological trauma, general anesthesia, infections. Some patients had a psychiatric, non-standardized, clinical assessment by a psychiatrist documenting the presence or absence of a comorbid psychiatric disorder.

During the neurological examination and physiotherapy treatment, we assessed the presence of any trigger factor which induced or exacerbated their FMD symptom. Patients were asked about triggers including visual (light), auditory (noise), tactile (self-touch), emotional (fear, low mood), movement/physical efforts. Only those triggers emerging during the clinical evaluation (history and/or neurological examination) and/or physiotherapy treatment (i.e. movement/physical efforts) were considered and evaluated. The presence of a visual trigger was evaluated by producing a flashing lights in the patients’ eyes or in taking off sunglasses indoors, in those patients reporting a high sensitivity to light. Auditory triggers were tested by reproducing a noise (i.e., clapping hands). Exercise movement trigger was explored by asking patients to perform a vigorous exercise, at higher intensity than usual, walking on the treadmill, climbing up and down several stairs or make various vigorous types of exercise with whole body. The emotional triggers were recorded when the patient reported a clear change of emotional state *preceding* FMD (i.e. sudden fear of falling). Touch triggers were recorded when FMD occurred after the patient touched them self or were touched by another person in a “triggering” part of the limbs, trunk or head. “Other” triggers provoking FMD were observed in relation to other factors not classifiable within the previous categorizations (e.g., cold air-conditioning, Table 2). Because of the lack of systematic prospective recording of these triggers when the patient was first seen, two independent movement disorders experts retrospectively reviewed the video-recordings of the neurological examination and physiotherapy treatment and confirmed the presence of such triggers. Patients from this cohort were categorized according to whether they displayed (T-FMD) or not (NoT-FMD) one or more triggers.

Patients were further categorized as having paroxysmal FMD or “persistent with paroxysmal variability”. Paroxysmal FMD were defined as conditions characterized by sudden episodes of FMD lasting for a brief but variable period of time (i.e. the symptom came and went multiple times a day or an hour or with episodes of several times a week) with periods during which the movement was noted by the patient to be absent [23]. Persistent with paroxysmal variability described patients who considered their FMD to be present most of the time but varying in severity.

### Statistical analysis

Data were expressed as mean ± standard deviation (SD) for continuous variables, counts, and percentages for categorical variables. We compared the groups using the Mann–Whitney *U* test for continuous variables and the chi-squared test or Fisher’s exact test (if ≤ 5 expected frequencies) for categorical variables. Statistical analyses were performed using SPSS statistical software (version 20; IBM-SPSS, Armonk, NY, USA).

## Results

The study population was 100 patients with FMD; the mean age at onset was 40.9 ± 15.4 years; *n* = 82 (82%) were women. The data from 13 patients were excluded from analysis because video recordings and/or detailed clinical information were missing. Forty-three (43%) patients had a psychiatrist assessment. Of these, 22 (51%) patients did not receive a definite diagnosis and 21 (49%) received one or more psychiatric diagnoses. Data on phenotype, non-motor symptoms, comorbidities and precipitating factors (events at onset only) are shown in Table 1.

**Table 1:** Clinical and demographical features of FMD patients without and with triggers

Variable	Total (*n* = 100)	NoT-FMD (*n* = 12)	T-FMD (*n* = 88)	Group comparison
Sex, female, no. (%)	82 (82)	11 (91.6)	71 (80.7)	1.000^F^
Age, years, mean (SD)	41.4 (16)	50 (14.4)	39.7 (15.2)	0.016^ M^
Time since onset of symptoms to FMD diagnosis, years, mean (SD)	4.2 (4.7)	5.3 (6)	4.1 (4.5)	0.673^ M^
FMD phenotype, no. (%)				
Weakness	89 (89)	11 (91.7)	78 (88.6)	1.000^F^
Gait disorders	76 (76)	6 (50)	70 (79.5)	0.035^F^
Tremor	69 (69)	7 (58.3)	62 (70.5)	0.507^F^
Dystonia	34 (34)	2 (16.7)	32 (36.4)	0.213^F^
Jerks	21 (21)	2 (16.7)	19 (21.6)	1.000^F^
Facial movement disorders	19 (19)	1 (8.3)	18 (20.5)	0.454^F^
Parkinsonism	4 (4)	0	4 (4.5)	1.000^F^
Self-reported non-motor symptoms, no. (%)				
Fatigue	78 (78)	9 (75)	69 (78.4)	0.723^F^
Pain	72 (72)	10 (83.3)	62 (70.5)	0.501^F^
Headache	56 (56)	5 (41.7)	51 (58)	0.286^C^
Anxiety	51 (51)	4 (33.3)	47 (53.4)	0.192^C^
Insomnia	41 (41)	5 (41.7)	36 (40.9)	1.000^F^
Depersonalization/derealization	33 (33)	2 (16.7)	31 (35.2)	0.327^F^
Panic attacks	24 (24)	2 (16.7)	22 (25)	0.725^F^
Neurological comorbidities, no. (%)	20 (20)	2 (16.7)	18 (20.5)	1.000^F^
Non-neurological comorbidities, no. (%)	45 (45)	4 (33.3)	41 (46.6)	0.387^C^
Psychiatric comorbidities, no. (%)	21 (21)	4 (33.3)	17 (19.3)	0.271^F^
Associated FND, no. (%)				
Sensory functional symptoms	59 (59)	9 (75)	50 (56.8)	0.350^F^
Functional seizures	24 (24)	2 (16.7)	22 (25)	0.725^F^
Visual functional symptoms	26 (26)	3 (25)	23 (26.1)	1.000^F^
Cognitive functional symptoms	60 (60)	7 (58.3)	53 (60.2)	1.000^F^
Fibromyalgia	15 (15)	1 (8.3)	14 (15.9)	0.687^F^
Functional bowel syndrome	12 (12)	1 (8.3)	11 (12.5)	1.000^F^
Precipitating factors, no. (%)				
Surgery	27 (27)	1 (8.3)	26 (29.5)	0.172^F^
Physical trauma	27 (27)	3 (25)	24 (27.3)	1.000^F^
Psychological trauma	28 (28)	3 (25)	25 (28.4)	1.000^F^
General anesthesia	20 (20)	1 (8.3)	19 (21.6)	0.451^F^
Infections	6 (6)	1 (8.3)	5 (5.7)	0.545^F^

**Fig. 1:**
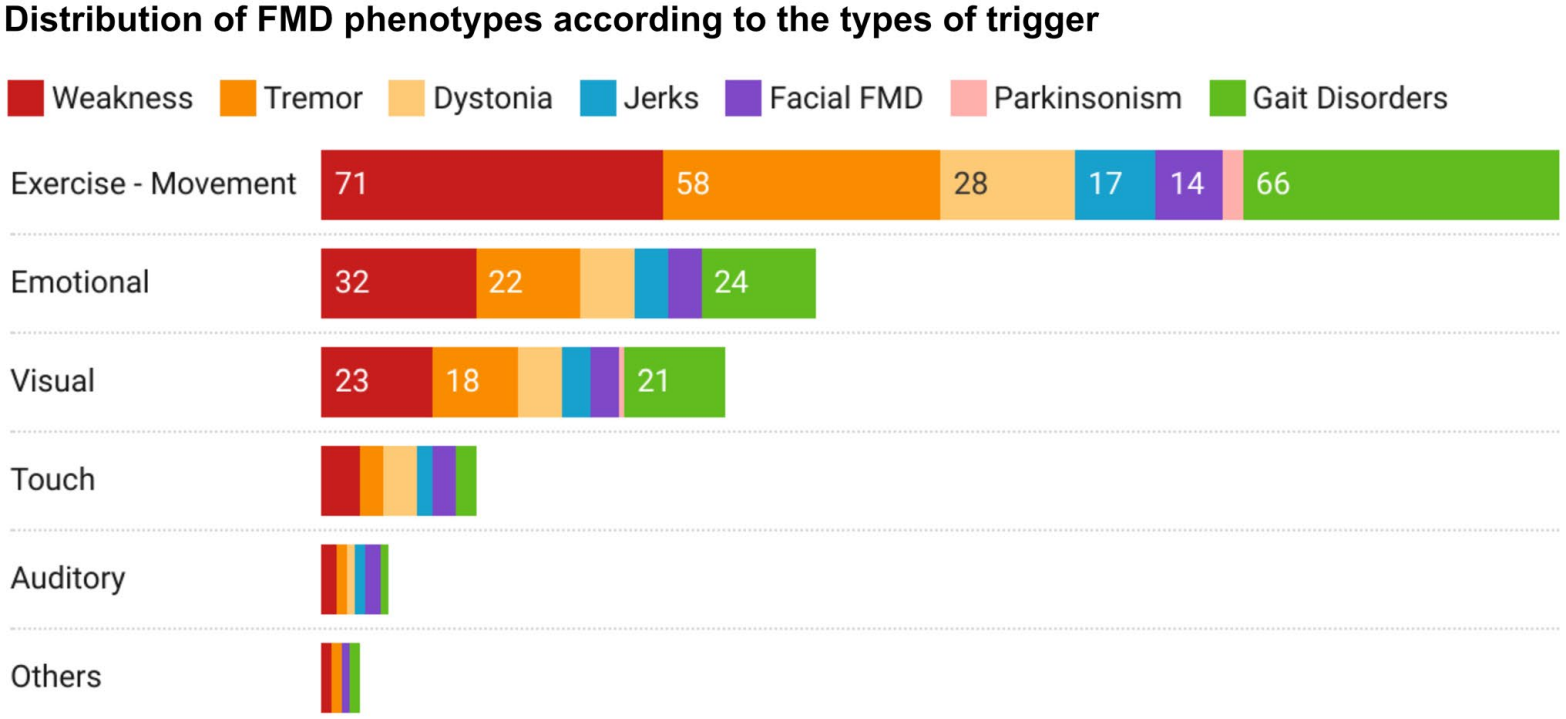
Distribution of FMD phenotypes according to the types of trigger

At least one trigger was noted in 88 patients (88%) and no triggers in 12 (12%). In those patients with trigger they were isolated in 39 (44%) patients and combined in 49 (56%). The most common type of trigger was exercise, followed by emotional, visual, touch, auditory and other stimuli (headache and cold temperature) (Table 2, Fig. 1). Videos, in the supplementary material, show examples of the different type of triggers.

**Table 2:** Frequency of different types of trigger inducing FMD

Exercise/movement (*n* = 79, 89.7%)	Exercise with whole body (sit-down/stand-up, from a chair squats) (*n* = 63, 79.7%), walking forwards or backwards (*n* = 33, 41.8%), lack of sleep (*n* = 32, 40.5%), climbing up and down stairs (*n* = 10, 12.6%), writing (*n* = 3, 3.8%), respiration (hypoventilation or hyperventilation) (*n* = 2, 2.5%), chewing (*n* = 1, 1.26%), swallowing food (*n* = 1, 1.26%), lifting forward or abduction upper limbs (*n* = 1, 1.26%)
Emotional (*n* = 33, 37.5%)	Anxiety (or worry) (*n* = 20, 60.6%), excitement/agitation (*n* = 15, 45.4%), fear (*n* = 14, 42.4%), self-image issue (self-doubt) (*n* = 12, 36.4%), panic attack (*n* = 7, 21.2%), stress (*n* = 6, 18.2%), anger (or frustration) (*n* = 3 9.1%)
Visual (*n* = 28, 31.8%)	Sensitivity to light (*n* = 26, 92.8%), flashing light (*n* = 1, 3.6%), watching a bicycle (*n* = 1, 3.6%)
Touch (*n* = 11, 12.5%)	Self-touch in the following body locations (*n* = 7, 63.6%): hairs, shoulder, neck, trunk, lower limbs; touch by neurologist and/or physiotherapist (*n* = 7, 63.6%)
Auditory (*n* = 4, 4.5%)	Noise (*n* = 4, 100%), clapping hands by someone (*n* = 1, 25%)
Others (*n* = 3, 3.4%)	Headache (*n* = 3, 100%), exposure to cold temperature (*n* = 1, 33.3%)

The triggers induced one or more FMD in various body regions: the lower (*n* = 77, 88%) and the upper limbs (*n* = 67, 76%), less frequently the trunk (*n* = 32, 36%), the face (*n* = 21, 24%) the head (*n* = 15, 17%), and the neck (*n* = 10, 11%). Forty-eight patients (54%) had triggers that induced FMD exacerbations or paroxysms lasting ≥ 5 min. A total of 77 patients reported triggers present at the onset and relapsing of FMD, while 11 were present only at relapsing of FMD. A total of 15 (17%) patients reported an overlapping between the precipitating psychological trauma and the emotional trigger. Our data however, only records triggers for people with an existing FMD.

**Fig. 2:**
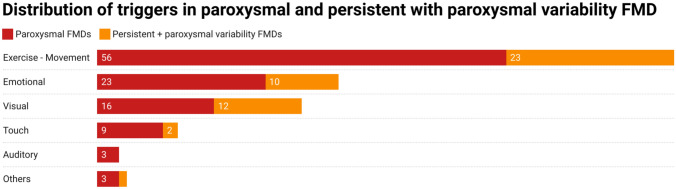
Distribution of triggers in paroxysmal and persistent with paroxysmal variability FMD

Patients with T-FMD were younger (*p* = 0.016) and had functional gait disorders (*p* = 0.035) more frequently than the NoT-FMD patients. No other statistically significant differences were found between the T-FMD and the NoT-FMD patients (Table 1). Paroxysmal FMD (74%, *n* = 65) were more frequent than persistent + paroxysmal variability FMD (26%, *n* = 23) in the T-FMD patients, while patients with persistent + paroxysmal variability FMD were more frequently noted to have a functional gait disorder (*p* = 0.033) and a visual trigger (*p* = 0.015) but less frequently dystonia (*p* = 0.028). No other statistically significant differences were found between the two groups (supplementary Table 1, Fig. 2).

## Discussion

In this study, we found that a high percentage of patients (88%) had a trigger which induced or exacerbated their FMD. Exercise movement was the most frequently observed trigger, followed by emotional, visual, touch, and auditory stimuli, all leading to a manifestation of FMD in various body parts, especially in the lower and the upper limbs. Moreover, we found no differences in the clinical and demographical variables between T-FMD and NoT-FMD, with the exception of younger age and more gait disorder [24] in the T-FMD group suggesting triggers don’t describe a discrete group of individuals with FMD [25].

Previous studies reported the occurrence of precipitating factors like physical events (i.e., injury, illness, general anesthesia) or psychological trauma/stressors (i.e. panic) at the time of symptoms onset [1–9]. These precipitating events, reported either alone or together with a history of predisposing factors (i.e., physical illness or exposure to illness in the family or personality traits), have not previously been clearly differentiated from triggers in patients where their FMD is already present. Triggers may potentially be a useful clinical clue in the diagnosis and the therapeutic management of FMD. Some patients may be hypersensitive to external stimuli (i.e., visual, tactile, auditory, motor) which may induce the sudden onset or exacerbate FMD [5, 12, 13, 15]. One study of 26 patients documented the presence of specific triggers in 50% of patients with paroxysmal psychogenic movement disorders. The triggers included increased stress (i.e. emotional), alcohol intake, walking (movement), loud noises (auditory), feeling frightened, sounds, and thirst [23]**.** Fasano et al. showed the effect of speech, eating and mouth movements on functional facial dystonia [26]. Individuals with functional jerks/myoclonus have commonly been noted to be startle responsive [13, 27], with some subtypes of this such as Latah being defined by response to startle [28]. Other studies reported the presence of abnormal eye and cranial movements provoked by examination (movement) [14, 15] or functional seizure induced by photic stimulation and saline injections [5] in patients with functional neurological disorders. Recently, Kramer et al. investigated the associations between negative affect and objective/subjective symptom levels of functional tremor and found a weak association, suggesting a poor relationship between daily stress and tremor symptom [29].

Many types of movement disorder have similar triggers such as exercise induced genetically determined paroxysmal dyskinesia, freezing in Parkinson’s disease related to visual stimuli such as doorways [18], or startle response in hyperekplexia. From a clinical perspective, triggers can be useful in the diagnosis of paroxysmal FMD. If a functional movement disorder is not present during examination, a trigger may help the clinician observe it to make a clinical diagnosis. From a therapeutic perspective, triggers can be important in understanding behaviour, especially avoidance, in relation to FMD. Multidisciplinary rehabilitation aims to improve normal movement patterns in FMD by ensuring the patient understands the nature of the condition, reducing abnormal self-directed attention, and promoting a sense of agency within a bio-psycho-social framework. Better awareness about the range and type of triggers could help clinicians and patients to better personalize a rehabilitation program. We find that some individuals with FMD avoid noisy or bright places because it increases the risk of developing abnormal movements, something that may be important in formulating a therapeutic plan. Identification of triggers forms a natural component of cognitive behavioural modification during psychological therapy. Triggers may also have more personal meaning, for example related to previous trauma involving touch, or injury, which may be important during psychodynamic approaches to therapy. Awareness of triggers could also be useful in physiotherapy, for example using triggers to bring out movements under controlled circumstances and using that experience to learn movement related desensitization and other techniques that promote automatic movement.

There have been few descriptions of paroxysmal FMD [23]. In this study we have taken a broader view of what a paroxysmal FMD is. Many patients classified as functional tremor or functional myoclonus present with intermittent movement disorder or movements that wax and wane through the day. Such patients are typically not described as paroxysmal FMD, a term usually reserved for individuals with isolated ‘attacks’ of movement disorder. Nonetheless much FMD is paroxysmal in this broader sense and a property that we consider is worth paying more attention to in terms of phenomenology and treatment.

Our findings are in line with a “predictive processing" explanatory model of FMD. Whereas precipitating events such as injury provide novel sensory information that can alter expectations and attentional focus about a certain symptom, triggering events would appear to operate at the level of classically conditioned responses created by that abnormal attentional focus [1]. Similar hypotheses have been proposed to describe the relationship between panic/autonomic arousal symptoms seen in panic and functional seizures [7] as well as functional drop attacks, where individuals have a symptom somewhere between a seizure and episodic paralysis [30]. There are clearly overlaps here to hypervigilance and startle response in post-traumatic stress disorder which is a common comorbidity of FMD and probably under detected in our sample.

It has been noted that for some patients with functional seizures feel that their attack creates some relief or avoidance of triggering sensations or thought [6]. Something similar may be operating in some patients with FMD as well. For example, facial dystonia triggered by photophobia that leads to involuntary contraction of orbicularis oculis could be seen as an adaptive phenomena to reduce the impact of that stimulus. Some patients with functional jerks note that the movement provides temporary relief from an ill-defined rising feeling beforehand, not dissimilar to that experienced by people with tics. The presence of transient ‘relief’ helps to explain how conditioned responses may sometimes occur, at an involuntary level, because of a reward response as well as a threat one.

We acknowledge several methodological limitations. We did not systematically prospectively evaluate all types of trigger for all patients, although did systematically record them retrospectively using video-recording during both the neurological examination and physiotherapy treatment. There are some kinds of triggers, especially FMD triggered by other symptoms such as headache, which are likely to be much more commonly found in a prospective study. With respect to precipitating events there is the problem of recall bias. We cannot rule out that patients may, for example, have overreported physical events and/or under reported life events at the onset of FMD. Our study does, however, provides novel data that brings together a range of clinical and prior observation to highlight that triggers are frequent in FMD and may have diverse, overlapping clinical presentations. Future prospective studies of FMD triggers could be an important avenue especially for developing more tailored rehabilitation interventions.

## Supplementary Information

Below is the link to the electronic supplementary material.

Supplementary file1 (DOCX 23 KB)

Supplementary file2 (MOV 18668 KB)

Supplementary file3 (DOCX 13 KB)
