# Correction to: Triggers in functional motor disorder: a clinical feature distinct from precipitating factors

**DOI:** 10.1007/s00415-022-11160-5

**Published:** 2022-05-16

**Authors:** Christian Geroin, Jon Stone, Serena Camozzi, Benedetta Demartini, Marialuisa Gandolfi, Michele Tinazzi

**Affiliations:** 1 Neurology Unit, Movement Disorders Division, Department of Neurosciences, Biomedicine and Movement Sciences, University of Verona, Verona, Italy. P.le Scuro 10, 37134 Verona, Italy; 2 Centre for Clinical Brain Sciences, University of Edinburgh, Edinburgh, UK; 3 Physiotherapy Bachelor, University of Milan, Milan, Italy; 4 Department of Neurosciences, Biomedicine and Movement Sciences, University of Verona, Verona, Italy

## Correction to: Journal of Neurology 10.1007/s00415-022-11102-1

The wrong Supplementary video was originally published. It has now been replaced with the correct Supplementary video.

The original article has been corrected.

