# Supplementary material for: Triggers in functional motor disorder: a clinical feature distinct from precipitating factors
Source: J Neurol. 2022 Apr 20;269(7):3892–8. doi: 10.1007/s00415-022-11102-1 (PMC9217842; doi:10.1007/s00415-022-11102-1)
Supplement: Supplementary file 1 — Supplementary file1 (DOCX 23 KB) [file 415_2022_11102_MOESM1_ESM.docx]

| **Supplementary Table 1. Clinical and demographical features of paroxysmal and persistent with paroxysmal variability FMD in patients with triggers.** | | | | |  |
| --- | --- | --- | --- | --- | --- |
| **Variable** | **Paroxysmal FMD**  **(n=65)** | **Persistent with paroxysmal variability FMD (n=23)** | **Group comparison** | | |
| **Sex, female, no. (%)** | 54 (83.1) | 17 (73.9) | .366^F^ | | |
| **Age, years, mean (SD)** | 40.7 (15.1) | 37.1 (15.5) | .416^M^ | | |
| **Time since onset of symptoms to FMD diagnosis, years, mean (SD)** | 4.4 (4.9) | 3.1 (2.5) | .620^M^ | | |
| **FMD phenotype, no. (%)** |  |  |  | | |
| Weakness | 57 (87.7) | 21 (91.3) | 1.000^F^ | | |
| Gait disorders | 48 (73.8) | 22 (95.7) | **.033**^F^ | | |
| Tremor | 48 (73.8) | 14 (60.9) | .241^C^ | | |
| Dystonia | 28 (43.1) | 4 (17.4) | **.028**^C^ | | |
| Jerks | 16 (24.6) | 3 (13) | .378^F^ | | |
| Facial movement disorders | 16 (24.6) | 2 (8.7) | .138^F^ | | |
| Parkinsonism | 3 (4.6) | 1 (4.3) | 1.000^F^ | | |
| **Self-reported non-motor symptoms, no. (%)** |  |  |  | | |
| Fatigue | 49 (75.4) | 20 (87) | .378^F^ | | |
| Pain | 44 (67.7) | 18 (78.3) | .340^C^ | | |
| Headache | 39 (60) | 12 (52.2) | .513^C^ | | |
| Anxiety | 35 (53.8) | 12 (52.2) | .890^C^ | | |
| Insomnia | 27 (41.5) | 9 (39.1) | .840^C^ | | |
| Depersonalization/derealization | 23 (35.4) | 8 (34.8) | .959^C^ | | |
| Panic attacks | 16 (24.6) | 6 (26.1) | .889^C^ | | |
| **Neurological comorbidities, no. (%)** | 15 (23.1) | 3 (13) | .380^F^ | | |
| **Non-neurological comorbidities, no. (%)** | 31 (47.7) | 10 (43.5) | .728^C^ | | |
| **Psychiatric comorbidities, no. (%)** | 14 (21.5) | 3 (13) | .542^F^ | | |
| **Associated FND, no. (%)** |  |  |  | | |
| Sensory functional symptoms | 34 (52.3) | 16 (69.6) | .151^C^ | | |
| Functional seizures | 16 (24.6) | 6 (26.1) | .889^C^ | | |
| Visual functional symptoms | 16 (24.6) | 7 (30.4) | .585^C^ | | |
| Cognitive functional symptoms | 40 (61.5) | 13 (56.5) | .673^C^ | | |
| Fibromyalgia | 13 (20) | 1 (4.3) | .102^F^ | | |
| Functional bowel syndrome | 9 (13.8) | 2 (8.7) | .721^F^ | | |
| **Precipitating factors, no. (%)** |  |  |  | | |
| Surgery | 22 (33.8) | 4 (17.4) | .137^C^ | | |
| Physical injury | 18 (27.7) | 6 (26.1) | .882^C^ | | |
| Psychological trauma | 19 (29.2) | 6 (26.1) | .774^C^ | | |
| General anesthesia | 17 (26.2) | 2 (8.7) | .138^F^ | | |
| Infection | 4 (6.2) | 1 (4.3) | 1.000^F^ | | |
| **Trigger, no. (%)** |  |  |  | | |
| Exercise | 56 (86.2) | 23 (100) | .104^F^ | | |
| Emotional | 23 (35.4) | 10 (43.5) | .491^C^ | | |
| Visual | 16 (24.6) | 12 (52.2) | **.015^C^** | | |
| Touch | 9 (13.8) | 2 (8.7) | .721^F^ | | |
| Auditory | 3 (4.6) | 0 | .564^F^ | | |
| Others | 3 (4.6) | 1 (4.3) | .675^F^ | | |
| Abbreviations: FMD, functional motor disorders; FND functional neurological disorders; SD standard deviation; statistical testing: Mann-Whitney U Test (M), Chi-squared test (C), Fisher’s exact test (F); statistically significant difference at p<0.05 shown in bold. | | | |  |  |
