# Supplementary material for: Triggers in functional motor disorder: a clinical feature distinct from precipitating factors
Source: J Neurol. 2022 Apr 20;269(7):3892–8. doi: 10.1007/s00415-022-11102-1 (PMC9217842; doi:10.1007/s00415-022-11102-1)
Supplement: Supplementary file 3 — Supplementary file3 (DOCX 13 KB) [file 415_2022_11102_MOESM3_ESM.docx]

# LEGEND TO THE VIDEO

Video 1. Movement exercise trigger. A 27-year-old woman, with a 48-month history of paroxysmal FMD. Physical activity (Sit-down/stand-up from a chair) triggered intermittent leg tremor and weakness. The patient could feel a premonitory urge**,** as she mentions, before the FMD onset.

Video 2. Emotional trigger. A 44-year-old woman with a 60-month history of paroxysmal FMD. The patient, seated in a wheelchair, reported she was unable to move her legs and walk, even supported by two physiotherapists, because of thoughts triggered by the fear of losing sensibility in her legs and falling. The second part of video shows that patient was able to move her legs and stand up straight while washing the dishes.

Video 3. Visual trigger. A 36-year-old woman with a 72-month history of paroxysmal FMD. Turning the light on triggered intermittent tonic spasms of the upper face, predominantly the right eyelids (blepharospasm) and the forehead. A left sided downward deviation of the mouth angle involving the perinasal region is evident in the lower face.

Video 4. Touch trigger. A 47-year-old woman with a 36-month history of paroxysmal FMD. A light slap on the left hemiface triggered tonic left-side spasms, resembling dystonia, involving the lips, eyelids, perinasal region, forehead, tonic jaw deviation, neck flexion and dystonic posturing of the arms and the trunk. A light slap on the right hemiface triggered contralateral dystonic posturing.

Video 5. Auditory trigger. A 47-year-old woman with a 12-month history of paroxysmal FMD. The sound of the physiotherapist clapping hands triggered paroxysmal jerks involving the head, the neck, and the shoulders, and grunting.

Video 6. Other triggers (cold air). In the same patient (Video 5), exposure to cold air-conditioning triggered variable episodes of similar FMD.
